# Processing and Characterization of Spent Nickel–Metal Hydride Type AA Batteries to Recover Valuable Materials (Cobalt, Nickel and Rare Earth Elements)

**DOI:** 10.3390/ma17194908

**Published:** 2024-10-07

**Authors:** Gheorghe Iacob, Valeriu-Gabriel Ghica, Florentina Niculescu, Mircea-Ionuţ Petrescu, Ana Vasile

**Affiliations:** 1 Department of Engineering and Management of Metallic Materials Production, National University of Science and Technology Politehnica Bucharest, 313 Splaiul Independentei, J Building, 60042 Bucharest, Romania; gheorghe.iacob@upb.ro (G.I.); ipetrescu@yahoo.com (M.-I.P.); 2 Doctoral School of the Materials Science and Engineering Faculty, National University of Science and Technology Politehnica Bucharest, Splaiul Independenţei 313, 060042 Bucharest, Romania; anavasile01@yahoo.com

**Keywords:** spent Ni-MH batteries, materials recovery, high metal content, characterization

## Abstract

The experimental research was focused on the investigation of valuable material from spent Ni-MH type AA batteries, namely the metal grid anodes and the black mass material (anode and cathode powder). The materials of interest were analyzed by X-ray fluorescence spectroscopy (XRF), ICP-OES (inductively coupled plasma optical emission spectrometry), optical microscopy, scanning electron microscopy (SEM), energy-dispersive X-ray analysis (EDX), electron backscatter diffraction (EBSD), and X-ray diffraction (XRD). The analyzed grids have a high Fe content, but some of them correspond to the Invar alloy with approx. 40% Ni. In the black mass material, round particles and large aggregations were observed by SEM analysis, showing a high degree of degradation. The XRD analysis reveals the presence of only three compounds or phases that crystallize in the hexagonal system: La_0.52_Ce_0.33_Pr_0.04_Nd_0.11_Co_0.6_Ni_4.4_, Ni(OH)_2_, and La_5_Ni_19_. The obtained results provide useful and interesting information that can be used for further research in the recycling and economic assessment of metals from spent Ni-MH batteries.

## 1. Introduction

In Europe, 27,000 tons of used batteries are recycled, while more than 200,000 tons of portable batteries are sold annually on the EU market. According to a study conducted by a local company, 40 million portable batteries are sold annually in Romania [1], which, after use, although considered hazardous waste, are mixed with household waste or incinerated. The average collection rate in European countries is 13%, and half of this percentage is achieved by only five countries, among which Belgium stands out, which annually collects and recycles more than 50% of batteries used. Poland has so far only reached 1% of the proposed collection target [2,3].

As the demand for batteries has increased over time around the world, the risks of pollution have also increased; therefore, collection and recycling methods must be continuously improved. The need to collect and recycle batteries varies from one country to another, but there is a definite trend towards stricter control over this aspect [4].

The recovery of useful materials from used batteries is an extremely versatile field with countless research projects classified according to the type of batteries:

- Primary or disposable batteries;
- Secondary batteries that can be recharged and reused.

Secondary batteries come in a series of varieties, starting from the classic Pb-acid type battery up to the NiCd, Ni-MH, Li-ion, LiPo, and NaS types, and the approach methods are diverse and continuously being developed [5,6,7]. This is why many researchers are in a continuous rush to find the key to success, namely a simple, efficient recovery technology with a high recovery yield and that is, above all, economical from all points of view (results in recovered products with a higher value through higher extraction selectivity).

The Ni-MH battery is a secondary power source, and it consists of two components (electrodes):

- Anode—nickel/lithium hydride or nickel/lanthanum.
- Cathode—nickel oxide.

The best electrolyte used to power the system is potassium hydroxide. This is an alkaline power source according to modern classification [8].

The chemical reactions that occur between the two electrodes of the Ni-MH battery are shown below [8]:

Anodic reaction:

MH + OH^−^ ↔ M + H_2_O + e^−^ (1)

Cathodic reaction:

NiO(OH) + H_2_O + e^−^ ↔ Ni(OH)_2_ + OH^−^ (2)

Global reaction:

NiO(OH) + MH ↔ Ni(OH)_2_ + M (3)

The anode is a hydrogen-absorbing composition. It can absorb a large amount of hydrogen; on average, the amount of absorbed element can exceed the volume of the electrode by 1000 times. To achieve complete stabilization, lithium or lanthanum is added. During the charging process, the hydrogen atom dissociates from Ni(OH)_2_ being absorbed by the MH alloy; then, during the discharge process, another hydrogen atom dissociates, but this time it dissociates from the MH alloy and combines with NiOH, thus forming Ni(OH)_2_ [9].

Ni-MH batteries are still among the best secondary batteries in the world, due to both their excellent electrical performance and minor environmental effects compared to lead–acid and nickel–cadmium batteries [10,11], and even if new types of batteries appear periodically, they maintain their position on the market [6,7,10], with a share of approx. 28% after Li-ion-type batteries, which have a share of 37% [12], and their consumption is rising because of the global increase in the use of electronic devices. On average, the maximum life cycle of these batteries is approx. 1000 cycles, which means that substantial amounts of used Ni-MH are discarded after 3–5 years of useful life [13,14,15].

In addition, in just a few decades, huge demand growth, increasingly strict mining regulations, and supply chain fragilities will lead to shortages in both nickel and cobalt and especially rare earth elements (REEs), which are critical for Ni-MH preparation [16,17]. Powering electric vehicles with Li-ion batteries and innovative technologies for energy storage from renewable sources cannot be achieved without the vital contribution of nickel, which plays a key role [18,19].

Given that Ni-MHs are rich in nickel (36–42%), cobalt (3–5%), and REEs (5−25%), these waste streams are increasingly being investigated as important potential resources for Ni, Co, and REEs [11]. Nickel is a high-value metal of key strategic industrial importance. The evolution of the nickel market is closely related to the evolution of stainless-steel production. Of the world’s nickel production, 70% is consumed in the production of stainless steel. In the waste sources being targeted, it is present as Ni salts in a mixture with other metals and materials from which it must be separated to recover high-purity, valuable Ni metals or Ni metal salts [20]. Cobalt and lanthanides (rare earths) are further critical strategic metals to be recovered from this source [21,22].

A significant proportion of spent batteries are still used in pyrometallurgical processes to produce other types of materials like ferro-nickel or ferro-nickel-molybdenum, which means that most of the useful elements are recovered, but a certain amount is lost following the refining processes of the resulting melts and their conversion into slag, which later is stored, or a small part is transformed into other products [22,23].

If the nickel and iron in batteries can easily find new uses through pyrometallurgical routes (SNAM-SAVAN, SAB-NIFE, and INMETCO industrial processes), the same can not be said about cobalt and rare metals [23,24,25]. The extraction and processing of these metals are still sensitive subjects; their price has been in a continuous increase period for a long time, making their recovery and valorization necessary.

Currently, hydrometallurgical processes—material dissolution using reagents and selective precipitation, solvent extraction crystallization, and other chemical operations for the recovery of dissolved metals—offer a promising alternative for the sustainable recycling of used batteries, to the detriment of pyrometallurgical routes, which are often associated with high energy consumption, significant losses of valuable metals, and hazardous gas emissions [12,14,16,20,21,23,25].

Unlike Li-ion batteries, Ni-MH active anode materials are more like hydrogen storage materials and tend to be composed of highly reducing sub-states such as hydrogen-absorbing alloys, alloys that are typically based on misch metals (La, Ce, Pr, and Nd), Ni, Co, and Mn [26,27]. Typical technologies used to recycle spent Ni-MHs involve H_2_SO_4_ leaching followed by REE precipitation and then solvent extraction to separate the other metals [12,14,20,21,28,29]. The processing of spent Ni-MHs requires the introduction of a reagent with excellent oxidation performance as well as a precipitation reagent (Na_2_SO_4_) to capture REEs [12].

This work presents the stages of obtaining some intermediate products from used Ni-MH batteries by using simple physical–mechanical methods which are easy to apply on a larger scale. The characterization of the materials contained in these types of batteries (black mass material and metal grid anodes) with high contents of nickel, cobalt, and rare metals was conducted through detailed XRF, SEM-EDX, BSED and XRD analyses to find the elements contained and characterize the material specific to each category of interest. This study will stand as the starting point for initiating an optimal process for extracting valuable metals.

## 2. Materials and Methods

### 2.1. Processing of Spent Nickel–Metal Hydride Batteries

**Figure 1:**
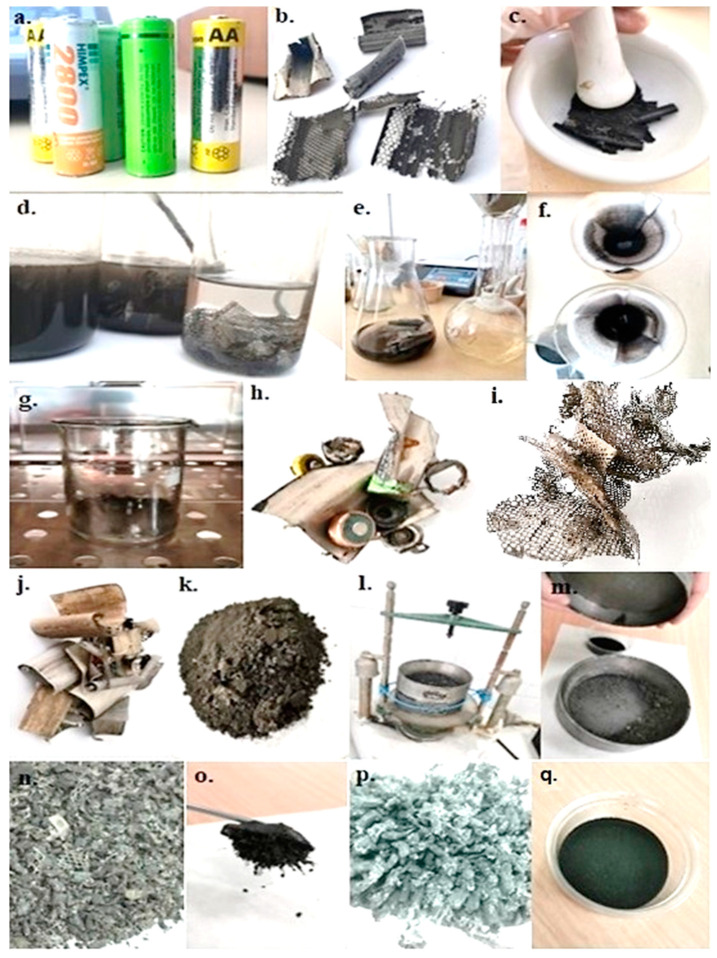
Processing of Ni-MH batteries: (**a**) before dismantling, (**b**) after dismantling, (**c**) crushing the mixed material, (**d**) washing with distilled water, (**e**,**f**) the filtration process, (**g**) oven drying the powder, (**h**) metallic casings, (**i**) grids, (**j**) non-metallic fraction after drying, (**k**) powder after oven drying, (**l**,**m**) sieving, (**n**) the coarse product obtained after sieving (mixture of pieces of metal grids with mixed content and non-metallic fraction), (**o**) magnetic separation, (**p**) pieces of magnetically extracted metal grids, (**q**) final powder.

The batteries used for the experimental research were of the Ni-MH type AA (re-chargeables/secondary batteries) from Duracell 2450 mhA (Duracell Inc., Chicago, IL, USA), Hama 2700 mhA (Hama GmbH & Co KG, Monheim, Germany), and Tronic Energy 2100 mhA (Tronic Technology Global Ltd., Shenzhen, Hong Kong). They have been used for a period of 4 years, having undergone more than 300 charge/discharge cycles. Figure 1 shows the stages of their processing to obtain the part materials.

The processing of used Ni-MH batteries was carried out manually by cutting, then separating and sorting into various fractions (metallic and non-metallic). In most studies regarding the recycling of Ni-MH batteries, a separation is made of the two categories of anode and cathode materials and their subsequent characterization. In our research, we chose the material recovered and analyzed to be the mixture of anode and cathode, because if we want to implement a process on an industrial scale, it is quite difficult to achieve, if not almost impossible, the separation of the two categories and the selective recovery of valuable metals. Thus, we can have a clear look at the material and the part elements, and we can establish an optimal recovery and recycling strategy.

After this stage, the recovery of the following categories of materials resulted, on which some technological operations were applied, and they were later characterized physico-chemically, morphologically, and structurally. These categories are as follows:

- Non-ferrous material (metal grid anodes): 4.13 wt.%.
- Ferrous material (metallic casings): 17.95 wt.%.
- Black mass material (anode and cathode powder): 41.91 wt.%.
- Coarse material (metal grid parts with embedded powder content): 26.21 wt.%.
- Non-metallic material (plastic polymers—insulators, separators, paper): 3.66 wt.%.

The black mass material was weighed before and after oven drying to figure out the moisture content of the batteries, which was determined to be 3.98 wt.%. The loss of material throughout the battery processing and sample preparation was also found (by the difference between the total quantity and the sum of all quantities obtained), and this was 2.16 wt.%.

**Figure 2:**
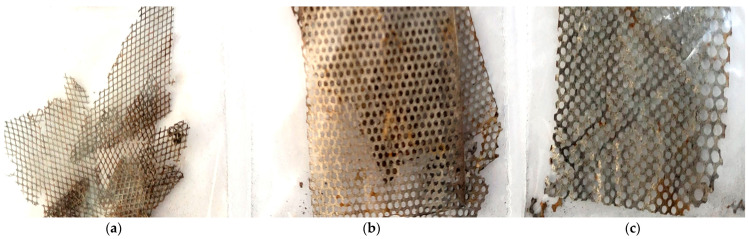
Images of the metal grids found in processed Ni-MH batteries: (**a**) type 1 (T1), (**b**) type 2 (T2) and (**c**) type 3 (T3).

The material caught on the metal grids was manually removed and added to the amount of black mass material. The metal grids and the coarse product obtained after sieving (Figure 2a), which consists of a mixture of pieces of metal grids and anodic and cathodic powder, were washed with distilled water several times to release the contained powder, with the metal parts later being easier to pick up. For future research, we thought of using a faster process with better results, such as the ultrasound process. It can provide a deeper cleaning of the metal grids [30].

### 2.2. Sample Characterization

The powder samples were chemically and quantitatively analyzed using X-ray fluorescence spectrometry as a non-destructive analysis technique, using an X-ray fluorescence spectrometer with wavelength dispersion (WDXRF) of the SPECTRO XEPOS type (SPECTRO Analytical GmbH, Kleve, Germany), by the TurboQuant-Alloys method. Also, the powder was examined by inductively coupled plasma optical emission spectrometry (ICP-OES) due to its superior sensitivity and broader elemental coverage using an Optima 5300DV spectrometer (PerkinsElmer Inc., Shelton, CT, USA). A Genius 5000 portable X-ray fluorescence spectrometer (Skyray Instruments Inc., Dallas, TX, USA) was used to find the chemical composition of the grids extracted from the batteries. This instrument allows the fast (2–60 s) and precise (minimum detection limits ~ 5 ppm) identification of a wide spectrum of elements (atomic number from 12 to 95 (element from magnesium (Mg) to uranium (U); it can be simultaneous analysis of 40 items)).

After disassembling the batteries, it was found that there are three distinct types of metal grids, with different shapes and sizes (Figure 2). To highlight their structure, a metallographic analysis was performed with the Optika B383 MET optical microscope, (Optika, Ponteranica, Italy) equipped with a digital camera, and image processing software. The microstructural characterization was conducted through scanning electron microscopy (SEM) FEI QUANTA 250 (FEI Company, Hillsboro, OR, USA), in high vacuum mode, and backscattered electron techniques (BSEs) using an angular backscattered detector. Point analysis, elemental mapping, and semi-quantitative analysis were performed through energy-dispersive X-ray spectroscopy (EDX) using the EDAX Element EDS Analysis System consisting of the ELEMENT Silicon Drift Detector Fixed and the ELEMENT EDS Analysis Software Suite (APEX™ 1.0, EDAX, Mahwah, NJ, USA).

X-ray diffraction investigations were conducted using an X’PERT MPD PANalytical X-ray diffractometer (PANalytical B.V., Almelo, The Netherlands) with horizontal Bragg–Brentano geometry and Cu Ka radiation. The structural data needed for comparison were taken from the ICDD (International Center for Diffraction Data), formally JCPDS (Joint Committee on Powder Diffraction Standards) files.

## 3. Results and Discussion

### 3.1. Characterization of the Metal Grid Anodes

#### 3.1.1. Chemical Composition

The chemical composition of the anodic grids, obtained after the XRF analysis of the three samples (T1, T2 and T3), is presented in Table 1.

The chemical analysis reveals the majority presence of Fe in each type of metal grid, followed by Ni with a significantly higher value for the T3 type of grid (approx. 40%) and Mo, which keeps a value of up to 2%. The presence of Co and Mn in reduced amounts is also seen. Thus, the chemical composition of the anodic grid T3 corresponds to the chemical composition of Invar alloys, notable for its uniquely low coefficient of thermal expansion and operation at extreme temperatures (−32 °C to 275 °C).

The other two types of grids (T1 and T2) were produced to lower the manufacturing price, by decreasing the Ni content at the same time with a slight increase in the content of Mn and Co compared to T3. Thus, the solution chosen by the producers was for these grids to be made of a nickel-plated mesh-shaped iron plate (perforated metal) that is coated with a metal hydride, such as Mm-Ni-Co (Mm = Misch metal), according to [31]. As can be seen in Figure 2, the majority presence of Fe in the basic composition leads to the degradation of the grids after immersion in distilled water and the initiation of the redox reaction.

#### 3.1.2. Optical Microscopy

**Figure 3:**
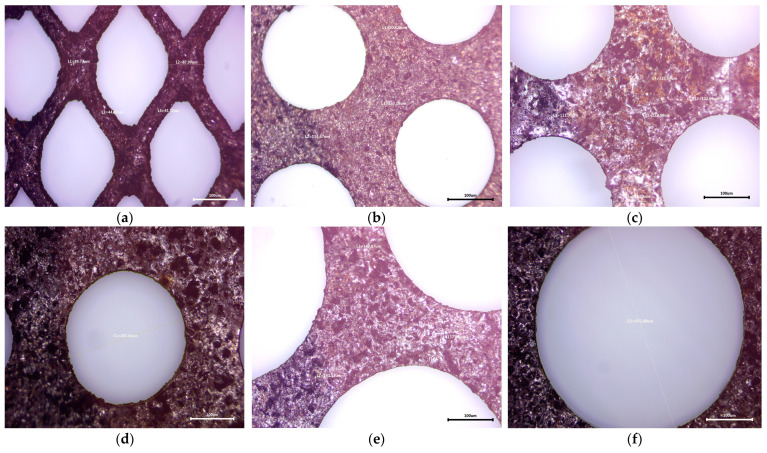
Optical microscopy images of metal grids: (**a**) anode grid (T1); (**b**) anode grid (T1); (**c**–**f**) anode grid (T3).

The metal grids used as anodes in Ni-MH batteries have different shapes and mesh sizes, as can be seen in Figure 2. The T1 and T2 types maintain their dimensions and shapes over the entire surface—the rhombus shape for T1 (Figure 3a) and the round shape for T2 (Figure 3b); the same can not be said about T3, which is formed by two sections with different mesh sizes (Figure 3c,d—section with small mesh sizes and Figure 3e,f—section with larger mesh sizes).

Since the batteries used in the experiments were completely exhausted, we cannot prove the Ni layer deposited on the steel sheets due to the chemical corrosion during the cathodic processes that led to the appearance of cavities on the sur-face and the significant degradation of the deposited layer. The inherent corrosion rate of the Ni-MH system is amplified by the presence of oxygen, which develops in overvoltage conditions, thus creating a passivating hydroxide layer that leads to low conductivity and slower electrode kinetics.

Thus, in addition to the changes in the composition of these metal grids, there are also changes in their shape and dimensions, most likely to compensate for the decrease in Ni content and the preservation of specific properties, by providing higher porosity with an extremely high surface area, leading to more reaction sites [32].

### 3.2. Characterization of the Black Mass Material (Anode and Cathode Powder)

The characterization of the material with valuable metal content (black mass) was conducted by X-ray fluorescence spectroscopy (XRF), scanning electron microscopy (SEM) and energy-dispersive X-ray analysis (EDAX).

#### 3.2.1. Chemical Composition

Following the determination of the chemical composition by XRF, the majority presence of the Ni element was found (81.29%), followed by Co (5.31%) and La (4.68%). Besides these, reasonable amounts of Ce and Ta were detected in the analyzed material, bringing the total concentration of rare earths to approximately 7% of the total mass of powder. Insignificant amounts of other elements were also detected, with their values being presented in Table 2, along with those discussed.

The material components determined by ICP-OES are presented in Table 3. Significant differences in the values obtained by the two methods are observed, as well as the presence of elements that were not considered in the XRF analysis, or that appeared in minor concentrations.

Ni is the predominant metal in the black mass material, and from the REEs group, La and Ce are the main metals, with minor contents of Pr, Ne, and Ta. Another aspect that we can note is the complexity of this material—20 chemical elements are shown in Table 3. This material, resulting from the processing of Ni-MH batteries, is of great interest, due to the high concentrations of the valuable metals Ni, Co, La, Ce, and Ta, so the economic aspect should not be neglected. Furthermore, an SEM-EDX analysis was performed, which completes the results of the XRF chemical analysis and ICP-OES.

#### 3.2.2. Scanning Electron Microscopy with Energy-Dispersive X-ray Analysis (SEM-EDX)

The material consisting of a mixture of anodic and cathodic powder (black mass) was subjected to detailed SEM-EDX and BSED analyses.

**Figure 4:**
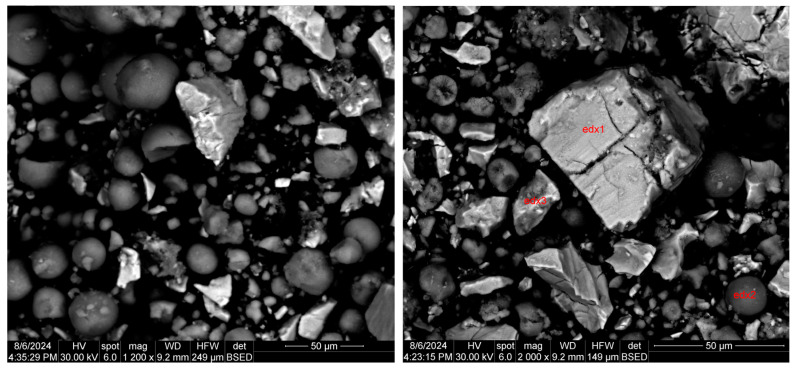
Representative SEM images of black mass material (anodic and cathodic powder).

The morphological aspect of the black mass is shown in Figure 4. Round particles of different sizes, large aggregations of particles of different sizes, and smaller pieces broken from larger aggregates (irregular particles) can be observed, most likely being formed because of the electrochemical processes inside the battery during the charge–discharge cycles and the hydrogen intercalation into the lattices of strong hydride-forming elements (i.e., La, Ce, Pr, and Nd). During the continuous charge–discharge cycles inside the battery, the pulverization of both the anode and cathode material takes place, leading to phase transitions that amplify the degradation process.

**Figure 5:**
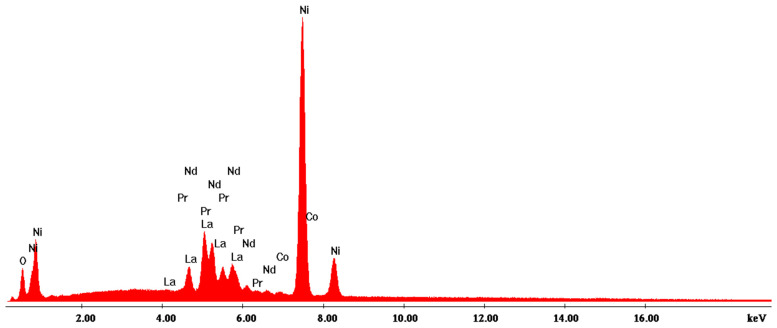
EDX1 spectrum associated with Figure 4.

**Figure 6:**
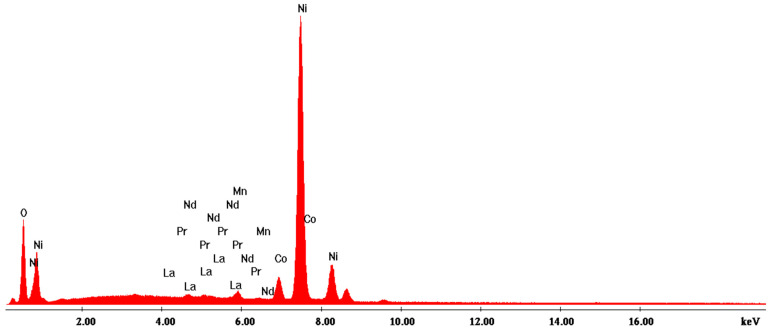
EDX2 spectrum associated with Figure 4.

**Figure 7:**
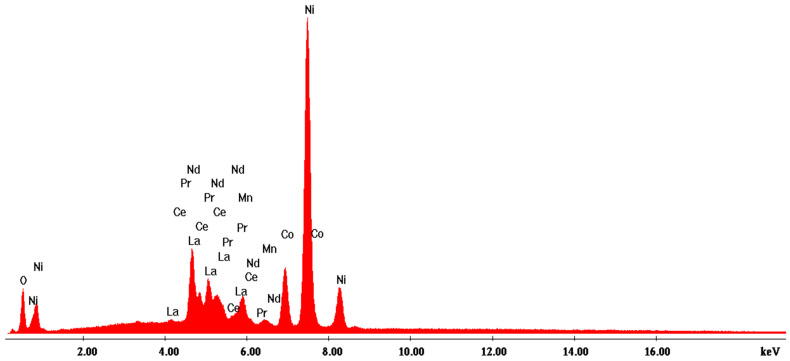
EDX3 spectrum associated with Figure 4.

According to the SEM image in Figure 4, three EDX analyses were performed on some predominant particles in the powder mass. The EDX spectra are shown in Figure 5, Figure 6 and Figure 7, and the micro-compositional values of the constituent elements are shown in Table 4, Table 5 and Table 6.

The EDX analysis in point 1—large aggregates—shows that Ni is found in the highest concentration (approx. 59%) and that there are high concentrations of Ce (13%) and Pr (13.4%).

The EDX analysis in point 2—round particles—shows that Ni is found in the highest concentration (approx. 69%) and that there are high concentrations of Co (approx. 5%) and exceedingly lesser amounts of La, Pr, Nd, and Mn.

Nickel hydroxide in spherical form is used in NiMH batteries to control the growth of particles. Nickel hydroxide has low conductivity, and to improve performance in batteries, a common procedure is the partial substitution of nickel ions with cobalt ions. Thus, the high concentration of cobalt and oxygen in Table 5 can be explained.

The EDX analysis in point 3—irregular particles—is the same as the other two previous analyses, namely that Ni is found in the highest concentration (approx. 52%). In addition to this, substantial amounts of La (16.3%), Ce (approx. 6.5%), Nd (3.2%), and Pr (approx. 2%) result from the analysis.

The presence of oxygen in high quantities, according to EDX analysis, reveals the fact that there are oxides of the elements in the powder. Oxidation of the elements can also take place due to their passivation, with the phenomenon being amplified by their powdery state and maintaining it in contact with air during processing and later until the time of the EDS investigations. The EDS analysis is performed on a thin surface of the analyzed particle (about 1 um and less), being extremely sensitive to any contamination, but this does not mean that the same concentration of oxygen is found in the entire volume of the sample.

**Figure 8:**
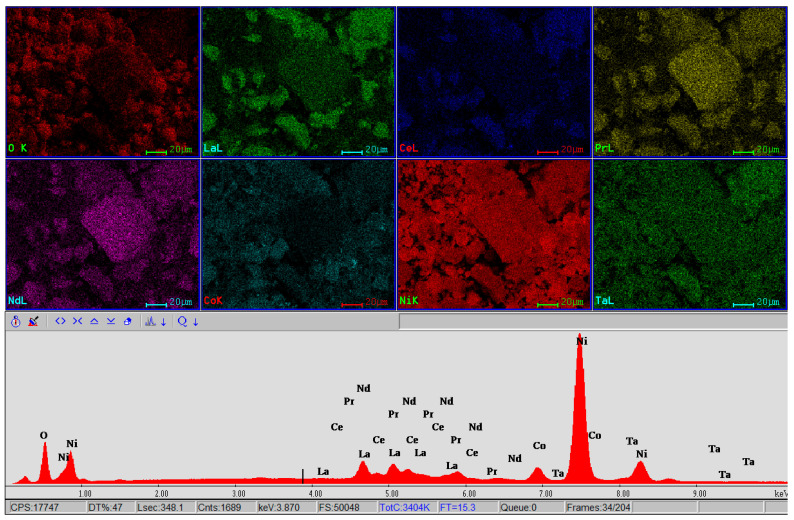
EBSD mapping and EDS analysis for the black mass material showing the distribution of elements O, La, Ce, Pr, Nd, Co, Ni and Ta in the respective area.

Figure 8 shows the EBSD-EDS mapping analysis of the black mass, which highlights the distribution of all the elements in the analyzed powder mass.

The EBSD mode of microscopy facilitates the observation of individual grain orientations, local texture, and the identification of phase distributions on the surfaces of polycrystalline materials, and the EDS spectrum obtained on the sample shows all the well defined spectral lines specific to the O, La, Ce, Pr, Nd, Co, Ni and Ta, with an adequate radiation background that facilitates a sensitive elemental analysis. According to the EDS spectrum, one can see the appearance of another essential element, Ta, which was not detected in any of the three EDX analysis points but appears in the XRF chemical composition. The presence of lanthanum, in the form of a compound, is also confirmed by XRD analysis.

#### 3.2.3. X-ray Diffraction Analysis (XRD)

**Figure 9:**
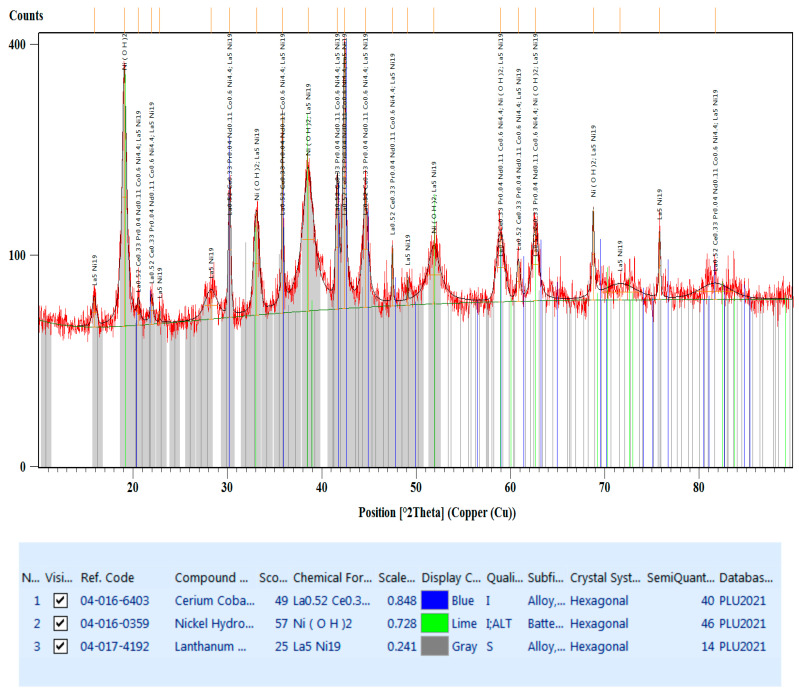
The diffractogram of the black mass material.

The diffractogram from Figure 9 shows the diffraction peaks of the compounds present in the black mass material (anode and cathode powder). According to this, there are three categories found:

- La_0.52_Ce_0.33_Pr_0.04_Nd_0.11_Co_0.6_Ni_4.4_, which crystallizes in the hexagonal system, space group P6/mmm (blue lines).
- Ni(OH)_2_, which crystallizes in the hexagonal system, space group P-3m1 (lime lines).
- La_5_Ni_19_, which also crystallizes in the hexagonal system but with a different space group P63/mmc (gray lines).

The XRD analysis helped to find the crystallographic compounds and if changes occurred during the life cycle of the Ni-MH batteries. This does not show the presence of foreign crystallographic phases, but only the β-nickel hydroxide phase. The result is consistent with [32].

In addition to this phase, the typical compounds AB_5_ are also found, in our case the La_0.52_Ce_0.33_Pr_0.04_Nd_0.11_Co_0.6_Ni_4.4_ compound, where A is the rare earth element (REE) mixture of La (lanthanum), Ce (cerium), Pr (praseodymium), and Nd (neodymium), and B is a mixture of Ni (nickel) and Co (cobalt). The recovery of this compound without the separation of the REEs and its reuse in the production of new Ni-MH batteries or H-storage portables [33] will reduce the cost of recycling by drastically reducing the energy required and the processing time. In [34], a compound of the REE*M_x_Ni_5-x_ type was successfully produced using solution combustion methods followed by a reduction process. The compound crystallizes in the hexagonal system, space group P6/mmm. The REEs in the form of oxalates were recovered from used NiMH batteries through hydrometallurgical processes and used to synthesize an REE*M_x_Ni_5-x_ (where x = 0.4) hydrogen storage material [34].

The negative electrode of rechargeable nickel–metal hydride (Ni-MH) batteries is produced from the intermetallic phase LaNi_5_, which reacts with hydrogen gas at ambient temperatures and above. The La-Ni binary system has been intensively studied in recent years, and its phase diagram has undergone successive changes, and the latest version shows eight intermetallic phases (i.e., La_3_Ni, La_7_Ni_3_, LaNi, La_2_Ni_3_, La_7_Ni_16_, LaNi_3_, La_2_Ni_7_, and LaNi_5_) [35]. In [36,37], the existence of the intermetallic phase La_5_Ni_19_, found between La_2_Ni_7_ (La-78.8 at.% Ni) and LaNi_5_ (La-83.2 at.% Ni), was discovered for the first time. The equilibrium phase is stable at elevated temperatures (1000 °C) but decomposes into La_2_Ni_7_ and LaNi_5_ below 900 °C and presents intergrowth structures that significantly improve the hydrogen absorption rate [38].

Most metals, when subjected to pressure, create a strong bond with hydrogen, thus resulting in metal hydrides; one powerful example is LaNi_5_H_6_. Key details on the content of metal hydrides in black mass can be obtained by deconvoluting associated XRD peaks and employing TPD analysis (temperature programmed desorption). TPD measurements can provide valuable information that helps to optimize the performance of catalysts but also to develop new materials with improved performance [39,40,41,42].

## 4. Conclusions

Ni-MH battery waste is a valuable source for many critical metals, such as nickel, cobalt, and rare earths, so an elevated level of collection and processing is necessary to recover and recycle important materials to maintain a sustainable production, limiting the consumption of chemicals and energy that are becoming increasingly expensive. Another positive aspect about spent Ni-MH batteries that must be considered is the fact that their components do not degrade completely during their lifetime, and they can be successfully used to produce new components for the same type of batteries or even for new applications.

The main purpose of the current study is to make a significant contribution to the physicochemical and structural characterization of the material contained in used Ni-MH batteries and to evaluate its reuse potential to develop specific treatment methods. The results of the chemical analysis performed on metal grid anodes show that there are three types of such grids, and some of them have low concentrations of nickel (from approx. 13% to 14%) compared to approx. 40% Ni of others. Also, an optical microscopy analysis was performed on them, which shows the degree of chemical corrosion during the cathodic processes.

Characterization of the black mass material (anode and cathode powder) was conducted by XRF, SEM-EDS, EBSD-EDS, and XRD analyses. The ICP-OES analysis showed the presence of high quantities of five rare earth metals from the lanthanoids category (La—6.13%, Ce—4.25%, Nd—1.73%, Pr—0.61%, and Ta—0.42%), confirmed by XRF and EDS analyses, so the economic aspect has significant importance. The SEM analysis shows that most REEs are found together with Ni in the form of micron-sized powder as large aggregations of particles of varied sizes or irregular particles. The XRD analysis does not indicate the presence of foreign crystallographic phases but does indicate the presence of the classic β-NiOH phase and two compounds less common in the specialized literature: the compound REE*M_x_Ni_5−x_ (where the REE is a mixture of La, Ce, Pr, and Nd and B is a mixture of Ni and Co) and the compound La_5_Ni_19_ stable at high temperatures.

Thus, the results obtained from the study provide detailed and useful information for further research about the technical aspects of recovering valuable metals from spent NiMH batteries. Research on the recovery of valuable metals (Ni, Co, and REEs) is ongoing; a combined hydro- and electrometallurgical process is used for the selective recovery of metals, and we are quite close to establishing appropriate electrolytic processing and extraction parameters for Ni and Co, with the results being promising.

## Figures and Tables

**Table 1:** Chemical composition (XRF) of the metal grids.

Chemical Composition (%)			
Element	Anode Grid (T1)	Anode Grid (T2)	Anode Grid (T3)
Fe	83.98	83.69	57.71
Ni	12.94	14.22	39.67
Mo	1.55	1.29	1.73
Mn	0.51	0.10	0.11
Co	0.47	0.37	0.33
Ti	0.23	0.12	0.12
V	0.14	0.04	0.18
Pb	0.08	0.10	0.05
Nb	0.02	0.02	0.03
Ta	0.02	0.01	0.02
Cr	0.02	0.02	0.02
W	0.01	0.01	0.01
Zr	0.01	0.01	0.02
Total	100	100	100

**Table 2:** Chemical characterization (XRF) of the black mass material.

Element (%)										
Na	Mg	Al	Si	Fe	Co	Ni	La	Ce	Ta	O.E. *
3.49	0.46	0.78	0.18	0.17	5.31	81.29	4.68	1.54	0.87	balance

* O.E.—other elements in insignificant quantities (S, K, Ca, Ti, Cu, Zn, Y, Mo, Sn).

**Table 3:** Chemical characterization (ICP-OES) of the black mass material.

Element (% (*w*/*w*))																			
Li	Na	Mg	Al	Si	S	K	Mn	Fe	Co	Ni	Cu	Zn	Cd	Ba	La	Ce	Pr	Nd	Ta
0.072	1.07	0.19	0.94	0.9	0.0214	0.36	0.89	1.14	4.78	74.17	1.52	0.27	0.0011	0.0021	6.13	4.25	0.61	1.73	0.42

**Table 4:** Elemental analysis associated with EDX1 spectrum.

Analysis	Elem	Wt. %	At %	K-Ratio	Z	A	F
EDAX ZAF Quantification (Standardless) Element Normalized SEC Table: Default	OK	8.21	29.39	0.0271	1.1506	0.2861	1.0020
	LaL	6.63	2.73	0.0634	0.8709	1.0571	1.0386
	CeL	13.07	5.31	0.1325	0.8906	1.0792	1.0547
	PrL	13.39	5.32	0.1373	0.8854	1.0873	1.0647
	CoK	0.18	0.17	0.0015	0.9912	0.8676	1.0000
	NiK	58.52	57.08	0.5354	1.0308	0.8875	1.0000
	Total	100.00	100.00				

**Table 5:** Elemental analysis associated with EDX2 spectrum.

Analysis	Elem	Wt. %	At %	K-Ratio	Z	A	F
EDAX ZAF Quantification (Standardless) Element Normalized SEC Table: Default	OK	23.24	52.99	0.0844	1.0915	0.3322	1.0023
	LaL	1.05	0.27	0.0106	0.8213	1.1204	1.0967
	PrL	0.35	0.09	0.0038	0.8406	1.1324	1.1384
	NdL	0.58	0.15	0.0064	0.8357	1.1359	1.1643
	MnK	0.85	0.57	0.0097	0.9320	0.9809	1.2412
	CoK	4.81	2.98	0.0447	0.9344	0.9935	1.0000
	NiK	69.12	42.95	0.6707	0.9705	0.9999	1.0000
	Total	100.00	100.00				

**Table 6:** Elemental analysis associated with EDX3 spectrum.

Analysis	Elem	Wt. %	At %	K-Ratio	Z	A	F
EDAX ZAF Quantification (Standardless) Element Normalized SEC Table: Default	OK	8.46	29.13	0.0292	1.1509	0.2994	1.0017
	LaL	16.33	6.47	0.1602	0.8703	1.0747	1.0488
	CeL	6.45	2.54	0.0651	0.8791	1.0854	1.0571
	PrL	1.77	0.69	0.0183	0.8901	1.0941	1.0642
	NdL	3.21	1.22	0.0336	0.8849	1.1008	1.0742
	MnK	2.73	2.74	0.0258	0.9868	0.8745	1.0928
	CoK	9.04	8.44	0.0789	0.9904	0.8816	1.0000
	NiK	52.01	48.77	0.4847	1.0298	0.9050	1.0000
	Total	100.00	100.00				

## Data Availability

The original contributions presented in the study are included in the article, further inquiries can be directed to the corresponding authors.
